# Biomaterials for Interbody Fusion in Bone Tissue Engineering

**DOI:** 10.3389/fbioe.2022.900992

**Published:** 2022-05-17

**Authors:** Han Zhang, Zhonghan Wang, Yang Wang, Zuhao Li, Bo Chao, Shixian Liu, Wangwang Luo, Jianhang Jiao, Minfei Wu

**Affiliations:** ^1^ Department of Orthopedics, The Second Hospital of Jilin University, Changchun, China; ^2^ Orthopaedic Research Institute of Jilin Province, Changchun, China

**Keywords:** 3D printing, interbody fusion cage, titanium, PEEK, absorbable material, growth factors, ceramic material, coating

## Abstract

In recent years, interbody fusion cages have played an important role in interbody fusion surgery for treating diseases like disc protrusion and spondylolisthesis. However, traditional cages cannot achieve satisfactory results due to their unreasonable design, poor material biocompatibility, and induced osteogenesis ability, limiting their application. There are currently 3 ways to improve the fusion effect, as follows. First, the interbody fusion cage is designed to facilitate bone ingrowth through the preliminary design. Second, choose interbody fusion cages made of different materials to meet the variable needs of interbody fusion. Finally, complete post-processing steps, such as coating the designed cage, to achieve a suitable osseointegration microstructure, and add other bioactive materials to achieve the most suitable biological microenvironment of bone tissue and improve the fusion effect. The focus of this review is on the design methods of interbody fusion cages, a comparison of the advantages and disadvantages of various materials, the influence of post-processing techniques and additional materials on interbody fusion, and the prospects for the future development of interbody fusion cages.

## 1 Introduction

With population aging, spinal degenerative diseases have become a health problem that cannot be ignored, with a lifetime incidence of >50% (Chong et al., 2016; Lim et al., 2019; May et al., 2019; Warren et al., 2020). In the spinal orthopedics clinic, when nerve compression persists for a long time and conservative treatment fails, decompression surgery has remained the first choice for the treatment of intervertebral disc degeneration, but interbody fusion is crucial for the treatment of impaired vertebral stability, failure of intervertebral disc surgery, spinal deformity and spinal stenosis. (Jain et al., 2016; Mena et al., 2016; Phan and Mobbs 2016; Assem et al., 2017; Iunes et al., 2020). The purpose of this operation is to decompress the nerve root, maintain the height of the intervertebral disc, and achieve bony fusion (Folman et al., 2003; Hoy et al., 2013; Hoy et al., 2019; Fan and Guo 2020). It means we need to convert fibrocartilage joints into hard and continuous bone segments (Smoljanovic et al., 2010). The autogenous iliac bone graft is the gold-standard method for spinal fusion (Vanek et al., 2012; Farrokhi et al., 2017). Harvested autologous bone slices have better osteoinductive properties compared to allogeneic bone grafts (Huang et al., 2014; Grgurevic et al., 2020). However, autologous bone grafts are limited by additional surgical trauma, donor site complications, and the inadequate autogenous bone supply (Sekerci et al., 2013; Fomekong et al., 2017). Fusion failure is often caused by cage loosening and related complications (5–35%), so choosing an appropriate interbody cage or not directly affects the success rate of surgery (Virk et al., 2017).

The ideal bone-substitute material should be characterized by favorable bone induction, good biocompatibility, suitable mechanical strength, and a high performance-to-price ratio (Warburton et al., 2019; Verma et al., 2020). At present, the commonly used materials for interbody fusion cages can be divided into metallic and non-metallic options (Park and Lehman, 2020; Verma et al., 2020). The most commonly used interbody cages are made of non-absorbent materials, such as titanium (Ti) and poly-ether-ether-ketone (PEEK). Ti alloy may cause implant subsidence and segmental instability due to its high elastic modulus, and the use of PEEK is limited due to its poor biocompatibility leading to chronic inflammatory responses (Guo et al., 2020). Absorbable materials include magnesium (Mg) and other polymer materials. With the mechanical strength weakens due to degradation, the materials may not be able to provide sufficient support and lead to the interbody fusion failure. Therefore, it is necessary to explore new materials that promote interbody fusion.

Three-dimensional (3D) printing is a new and widely popular technology (Egan et al., 2018; Burnard et al., 2020). Most importantly, 3D-printed cages can be customized for individual patients to achieve a high degree of implant-to-anatomy match. An appropriate interbody cage is designed to maintain the height of the intervertebral body and keep the upper and lower vertebral bodies stable. In addition, the internal porous structure made by 3D-printing technology can induce new bone ingrowth, which realizes the fusion of the upper and lower vertebral bodies to achieve biological stability (Zhang et al., 2020a). Osseointegration can be further improved by making surface modifications and the addition of drugs and growth factors (Park and Lehman, 2020). Coating is a commonly used surface-modification technology, and better biological activity and osteogenic ability can be obtained with coating materials such as Ti and hyaluronic acid (HA). Commonly used drugs include simvastatin (SIM) and strontium ranelate (SRR), which can improve the local microenvironment and promote osteogenesis in patients with osteoporosis. Growth factors, such as bone morphogenetic protein (BMP) and epidermal growth factor, can promote interbody fusion by regulating the expression of osteogenic genes. And we list all Experimental studies and Clinical studies in Table 1 and Table 2 respectively.

**TABLE 1:** Experimental studies published recently.

Study	Additive	Scaffold	Technique	Animal Model	Results
(Zhang et al.)	SIM/poloxamer 407 Hydrogel	Ti	3D-printing	Rhesus Macaque	Promoted bone ingrowth and spinal fusion
Liang et al. (2020)	—	HA/PA66	Infiltrating	Goat	Effectively improved intervertebral bony fusion
Rodríguez-Vázquez and Ramos-Zúiga (2020)	—	Ch/HAp	Freeze-drying	Rat	Bone regeneration in a solid and well-structured fusion was induced
Chu et al. (2019)	Iliac crest bone graft	CS/PEEK (4:6)	Fabricated by compounding and injection-molding	Goat	Induced highly effective bone fusion
Gunzburg et al. (2019)	Autologous bone	PEEK	Ti coating	Ovine	Nano-surfaced cages resulted in greater spinal stiffness changes
Lu et al. (2019)	—	Tantalum	3D-knitted	Rabbit	Showed good biocompatibility and osteocompatibility
Walsh et al. (2018)	—	PEEK	Coating	Sheep	Newly formed bone tracked along the thin Ti-coated surface
McGilvray et al. (2018)	—	Titanium	3D-printing	Ovine	Reduced range of motion and increased bone ingrowth and construct stiffness
(Kirk et al., 2017)	Iliac crest bone graft	PEEK titanium composite (PTC)	Hybrid additive manufacturing approaches	Ovine	PTC constructs demonstrated significant reductions in range of motion and a significant increase in stiffness compared to PEEK and PSP
Ren et al. (2017)	—	MAACP/HA/CS	—	Goat	Provided a good fusion effect, enough biomechanical stability, and close integration with the surrounding bone
Bae et al. (2016)	RhBMP-2	PEEK	—	Sheep	RhBMP-2 dose-dependent osteoclastic resorption is a transient phenomenon
Wheeler et al. (2016)	mesenchymal precursor cells (MPCs)	PEEK	—	Ovine	MPCs are an alternative to autologous for lumbar interbody fusion procedures

**TABLE 2:** Clinical studies published recently.

Study	Additive	Scaffold	Technique	Case Number	Results
Mobbs et al. (2019)	Allograft	TI	3D-printing	1	Rapid recovery with significant fusion effects
Zippelius et al. (2018)	—	TI	3D-printing	50	Implant is safe and led to a very high fusion rate
Mokawem et al. (2019)	—	SiCaP-packed TI	3D-printing	93	Provided excellent rates of solid fusion in TLIF and LLIF surgeries
Ohanisian and Dorsi (2019)	—	TI	3D-printing	1	Stimulates osteogenesis and enhances fusion with a remarkable improvement of symptoms
Arts et al. (2020)	—	TI	3D-printing	50	The fusion rates of porous titanium and PEEK with autograft were similar
Siu et al. (2018)	—	TI	3D-printing	30	The subsidence rate was lower compared to that of the PEEK implant
Krafft et al. (2019)	—	TI	3D-printing	1	The difficulties in deformity correction secondary to osteoporotic fractures were overcome

Accordingly, we wrote this review to address above issues. Review was retrieved in PubMed databases and Web of Science by the keywords: “interbody fusion”, “cage”. A total of 658 papers were picked, we excluded some earlier published research and clinical articles, and 171 articles were finally selected for this review according to the criteria of inclusion and exclusion. This review introduces the application of interbody fusion cages in bone tissue engineering in detail and focuses on the design methods of interbody cages, including 3D printing, porosity design. The advantages and disadvantages of interbody cages made of different materials, such as Ti, tantalum, Mg, PEEK, and ceramics, are compared and improved methods are proposed. The effects of material post-processing techniques on interbody fusion are summarized, including coating techniques, and the effects of the internal addition of SIM, BMP, and SRR on interbody fusion are discussed. In addition, the application challenges of different interbody cages and directions for further exploration are summarized.

## Strategies for Designing Interbody Fusion Cages

### Traditional Interbody Fusion Cages

Before an interbody fusion cage design is chosen, the anatomical location, surgical approach, and implant size need to be considered. The anatomical model may be obtained through a computed tomography (CT) examination, and the matching interbody fusion cage can then be designed accordingly (Duan et al., 2016; Rieger et al., 2017; Sen et al., 2019; Gkt et al., 2020). Combined with the CT data of the intervertebral disc, the computer-aided design software (CAD) with computer-aided manufacturing (CAM) technology was used to design the 3D model of the interbody cage, and then the fused deposition modeling (FDM) and selective laser melting (SLM) technology were used to print interbody cage (Chen et al., 2019; Bassous et al., 2019). The anatomical data of the target implant area include the left–right diameter, anteroposterior diameter, intervertebral height, and the angle between the upper and lower surfaces. In addition, the increase in intervertebral height caused by the destruction of the upper and lower endplates during surgery should also be considered. Undersized implants may cause subsidence, while oversized implants can cause damage to neural structures due to excessive compression (Feng et al., 2014; Girod et al., 2017; Zhang et al., 2020b). The angle between the upper and lower surfaces mentioned above suggests the postoperative physiological curvature of the spine. A proper angle in this context will keep the posterior longitudinal ligament relatively tight after implantation, preventing the posterior longitudinal ligament from going slack and compressing the nerve. Moreover, an optimal angle can also avoid overstretching of the anterior longitudinal ligament, which may cause vertebral instability. Due to the different stages of fusion and the individual differences of the target, the designed interbody cages have various shapes, such as a kidney shape (Madan and Boeree 2003). Joffe *et al.* obtained previous data through CT imaging and designed a corresponding cervical fusion cage, then carried out 3D printing and implantation in a canine cervical interbody fusion model. The implants were not ground or polished in this context because the rough surface structure increased the area of bone contact to facilitate bone growth (Joffe et al., 2019). A customized intervertebral device restores the intervertebral space and achieves a certain degree of interbody fusion (Joffe et al., 2019). In addition, by designing hollow cages, the implantation of autologous bone and other implants can be ensured to promote osseointegration. Walsh *et al.* designed an interbody fusion cage (measuring 4.5 mm high, 10 mm wide, and 20 mm long) with a central hole for transplant material injection, which was printed to fit the L4–L5 intervertebral space of sheep, providing support for clinical implantation (Walsh et al., 2018). The design of the fixed device cannot be ignored, and the nailboard system is the most commonly used method applied to fixation (Hah et al., 2020). Fixed device is generally used to maintain the stability of the intervertebral space for a better fusion effect (Caplan et al., 2020).

### Porous Structures

The interconnected micropore structure of an interbody fusion cage can improve the bone-conduction ability, which also benefits cell proliferation and differentiation and bone regeneration. The pore size of human cancellous bone is 500–600 μm (Lim et al., 2019). Considering porous scaffolds applied in bone tissue engineering, the porosity should be >50%, especially in the range of 65–80%, where the structure and elasticity modulus are similar to those of human trabeculae (Zhang F et al., 2018; Bai H. T. et al., 2020). The stress between the cage and the endplates decreases with increasing porosity, while the range of motion of the vertebral bodies decreases with increasing fusion rates of the upper and lower vertebral bodies. Previous experiments have shown that a pore size of 50–500 μm is beneficial for the adhesion, proliferation, and differentiation of osteoblasts (Zhang Z et al., 2018; Bai H. et al., 2020). In a comparison of goat interbody fusion models, McGilvray *et al.* found that the range of motion between the vertebral bodies of the animals using porous Ti interbody cages was significantly reduced and the stability of the interbody fusion was increased, indicating that it obtained a better fusion effect. The reason for this is that bone growth occurs in the cage’s microporous structure, resulting in a greater overall mechanical structural stability and more effective fusion (McGilvray et al., 2018). The holes, in addition to providing space for new bone to grow in, can be used for the injection of bioactive structures, such as BMPs, antibiotics, and bioceramics, as well as for post-processing for better fusion results (Schnitzer et al., 2020).

### Other Special Structures

**FIGURE 1:**
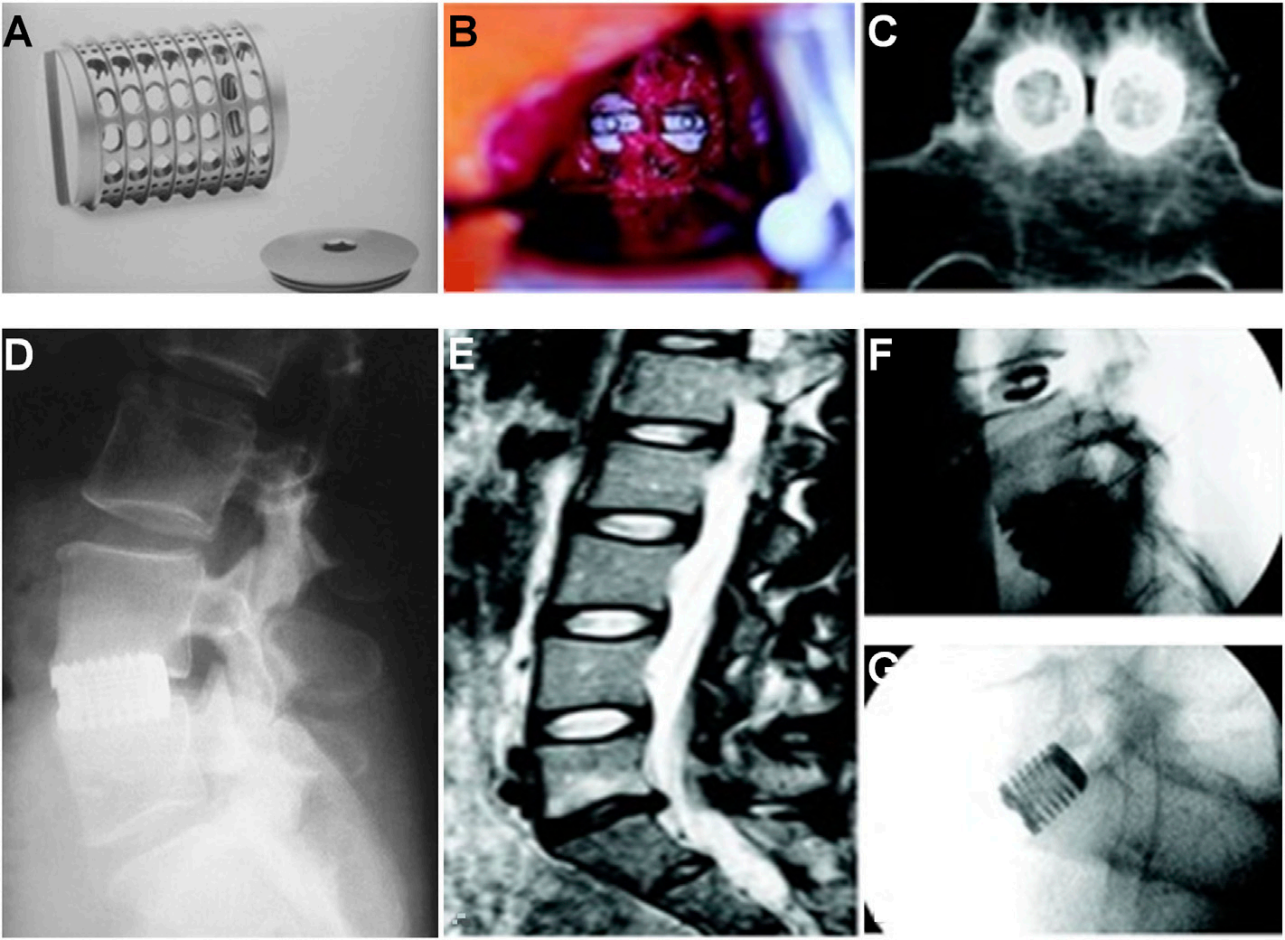
**(A)** INTER FIX threaded fusion device. **(B)** Intraoperative picture of the two cages implanted at the L5–S1 interspace. **(C)** Coronal CT scan 1 year postoperative. Bone has grown through and around the cages. **(D)** Lateral radiograph of INTER FIX. **(E)** Preoperative sagittal T2-weighted MRI with degeneration of the L5–S1 disc and a normal L4–L5 disc. **(F)** Discogram with morphologic abnormality of the L5–S1 disc and a large posterior anular tear. The L4–L5 disc is anatomically normal. Exact concordant 10/10 pain was reproduced at L5–S1. The L4–L5 injection caused no pain. **(G)** Postoperative lateral radiograph with INTER FIX cages in good position (Sasso et al., 2004).

**FIGURE 2:**
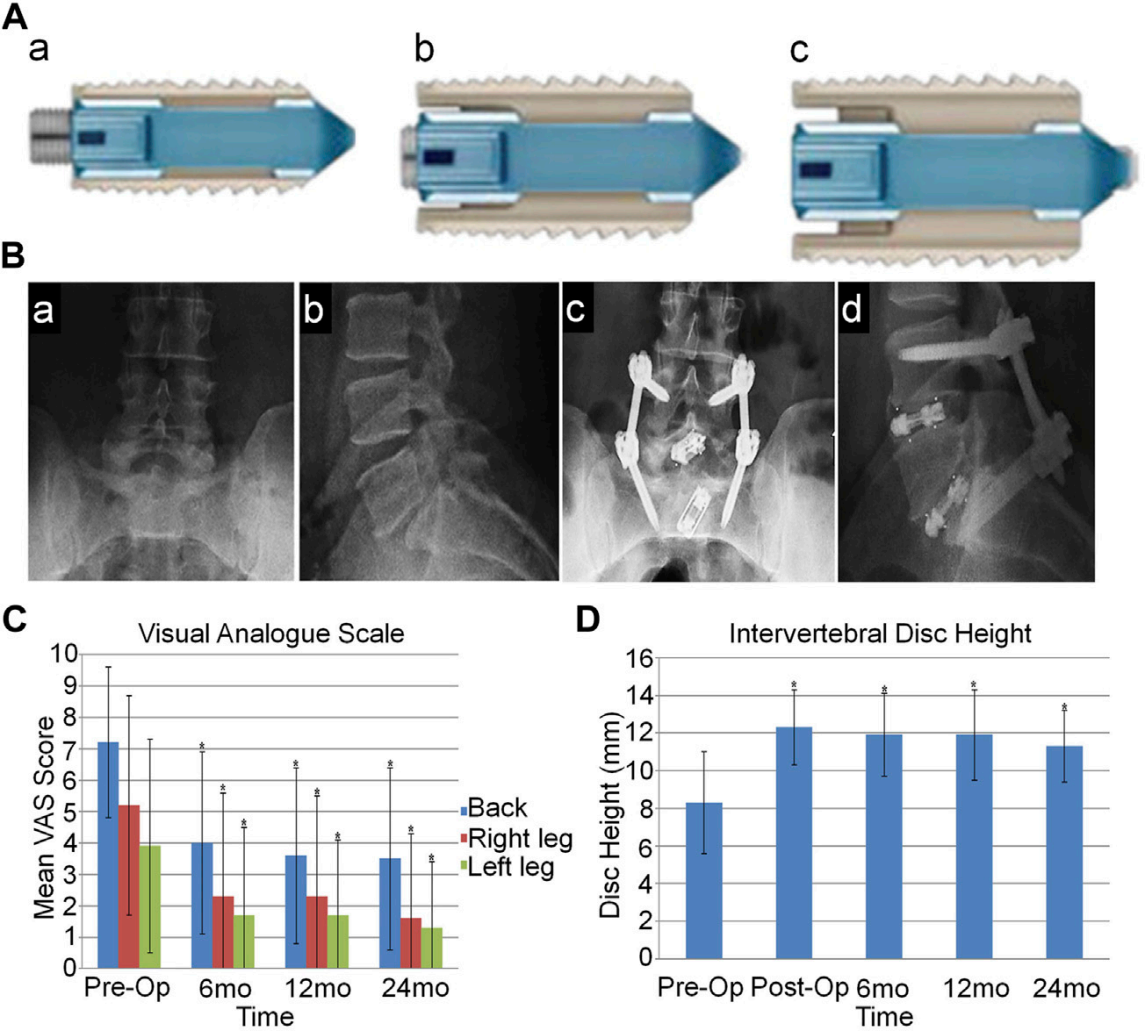
**(A)** Lateral view of the expandable interbody cage. The implant is shown in contracted **(A)**, expanding **(B)**, and fully expanded **(C)** conditions. **(B)** Preoperative anteroposterior **(A)** and lateral **(B)** plain-film radiographic images of the lumbosacral spine. Postoperative anteroposterior **(C)** and lateral **(D)** radiographic images of the lumbosacral spine demonstrating 2-level interbody reconstruction and transpedicular screw and rod instrumentation at L4eS1. **(C)** The bar graph shows visual analog scale scores for patients from the preoperative assessment through 24 months of follow-up. **(D)** The graph shows intervertebral disc heights of patients from the preoperative assessment through 24 months of follow-up (mean ± standard deviation values) (Kim et al., 2016).

In designing the interbody fusion cage, there are some special options available to facilitate certain tasks, such as reducing the difficulty of surgery or promoting the fixation of adjacent vertebral bodies by integrating the cage and the fixator. Sasso *et al.* designed a hollow threaded cylinder with a removable end cover that provides strength while reducing the weight and increasing the space available for the bone autograft. Each cylinder component has multiple holes for implanting the head and tail as well as small transverse holes to enhance the vascularization of the graft in the device. The matching internal fixation device is then designed correspondingly. A good fusion rate was obtained in patients implanted with this device (Sasso et al., 2004) (Figure 1). In addition, the threaded structure on the surface of the fusion cage can enhance interbody fusion and reduce the occurrence of postoperative complications, causing autogenous bone transplantation to rarely be needed (Hacker et al., 2000; Burkus et al., 2002). The screw trajectory design, planned screw length, and device matching the patient’s unique endplate anatomy should be considered when 3D-printing the threaded hole design to ensure uniform force on the endplate and device and to aid in fixation (Park et al., 2009; Caffrey et al., 2018; Easley et al., 2018). Mobbs *et al.* reported custom features with pre-angled threaded holes designed to enhance post-implantation fixation and endplate interfaces that match patient anatomy to ensure even loading on the endplates and device. Their patient recovered quickly after surgery, and their clinical symptoms were significantly improved (Mobbs et al., 2019). Bionic structures can also be considered. Zippelius *et al.* designed a “banana-shaped” cage made of Ti with curved rails on the surface that can help embed the endplates of the vertebrae, securing the cage to the desired location on the ventral edge strip (such as on the rails) while extending the surface and facilitating better skeletal integration, and they obtained a good fusion rate in 60 patients by using this approach (Zippelius et al., 2018). In addition, Kim *et al.* designed a scalable interbody device (inserted in the form of contraction and expanded *in situ* after being correctly located in the intervertebral space), which is suitable for minimally invasive interbody fusion. Clinical validation found that this device resulted in improved patient-reported clinical outcomes, recovery of disc height, and better fusion rates together with reduced risks of implant displacement, subsidence, rupture, and collapse compared to static devices (Kim et al., 2016) (Figure 2).

## 3 Interbody Fusion Cage Materials

### Ti and Its Alloy Material (Ti_6_Al_4_V)

Ti_6_Al_4_V is typically selected as the raw material for the manufacture of interbody cages because of its strength, excellent corrosion resistance, low density, biocompatibility, relatively low cost, and compatibility with magnetic resonance imaging (Dai and Jiang 2008; Knutsen et al., 2015; Burkus et al., 2017). The artifacts of Ti alloy generated on CT images are fewer than those associated with other metals, so the condition of the implant can be monitored after surgery (Joffe et al., 2019). 3D-printed porous Ti_6_Al_4_V, which can be prepared by selective laser melting or electron beam melting, has a low elastic modulus similar to that of cortical bone, so it is a promising orthopedic scaffold material (Liu et al., 2016; Walsh et al., 2019). Advances in 3D-printing technology have enabled the manufacture of Ti cages with complex internal geometries. Postoperative follow-up of 93 patients who received transforaminal lumbar interbody fusion (TLIF) and lateral lumbar interbody fusion (LLIF) using silicate-substituted calcium phosphate (SiCaP)-packaged 3D printing lamellar Ti cages was performed by Mokawem *et al.*, and their results demonstrated that the 3D-printed lamellar Ti cages packaged with SiCaP ensured excellent solid fusion rates during TLIF and LLIF surgeries (Mokawem et al., 2019). 3D printing of Ti makes prostheses rougher. Modifying the surface of an implant, such as its surface roughness and morphology, promotes bone consolidation and prevents the implant from falling off of the vertebral body (Mobbs et al., 2019). Ti implants are beneficial for bone growth because of their metal properties (i.e., valence charge), which attract neighboring proteins, cells, and body fluids (Cheng et al., 2020). In a previous study, however, Hauerberg *et al.* found that, for cervical radiculopathy caused by intervertebral disc herniation, there is no difference between simple discectomy and Ti cage fusion after discectomy (Hauerberg et al., 2008).

Ti has a high elastic modulus such that the porous Ti layer hardly deforms under compression during implantation or under physiological loading (Gunzburg et al., 2019). Arts *et al.* found that the fusion rate of porous Ti was similar to that of autologous bone–filled PEEK at 12 months during a prospective study of single-segment anterior cervical discectomy and fusion (ACDF) patients using 3D-printed porous Ti neck implants (Arts et al., 2020). Through a clinical comparison, Krafft *et al.* found that the sinking rate of 3D-printed porous Ti interbody fusion cages was significantly lower than that of PEEK interbody fusion cages (Krafft et al., 2020). 3D-printed lamellar Ti cages filled with SiCaP bone grafts can also achieve promising fusion rates in adult degenerative diseases by providing more consistent bone growth and biological fixation (McGilvray et al., 2018; Mokawem et al., 2019). However, the Ti_6_Al_4_V material has a high elastic modulus. Due to the different elastic moduli of cortical bone, the spinal fusion cage easily sinks, resulting in a decrease in intervertebral height, pedicle stenosis, and so on (Seaman et al., 2017). At present, through the use of 3D-printing technology, Ti_6_Al_4_V materials have been successfully used in artificial hip replacements, semi-pelvic replacements, and artificial vertebral replacements in patients with spinal tumors. The advantages of 3D-printing technology in creating medical Ti alloys are gradually being highlighted due to the abundant processing methods in play (Niu et al., 2019).

### Tantalum

**FIGURE 3:**
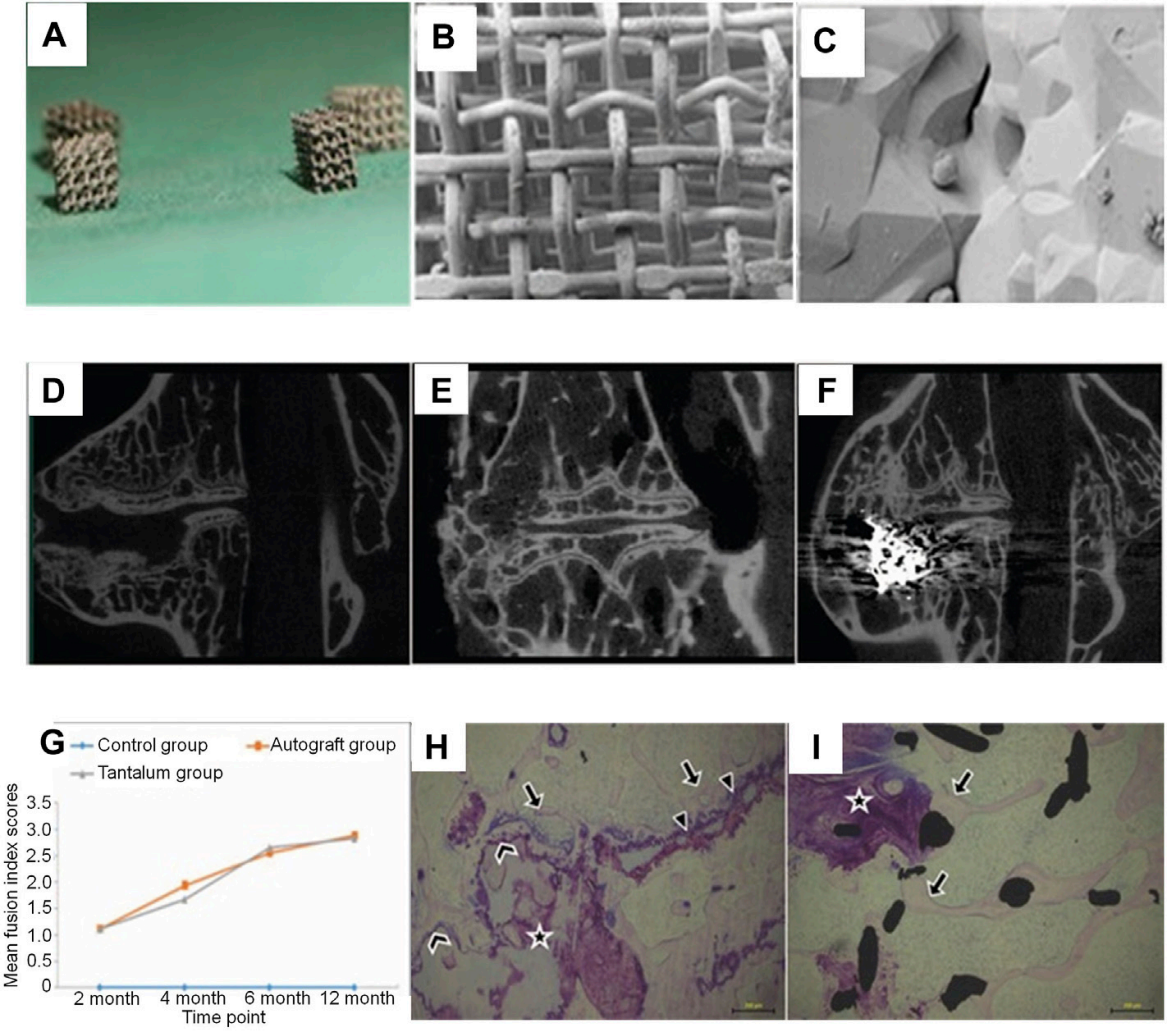
**(A–C)** The outlook of cubic porous tantalum implants (whose length, width, and height are 2.5–3.0 mm). Scanning electron microscopic images of porous tantalum taken at a lower magnification (B; 85×) and a higher magnification (C; 5,000×). **(D–F)** Micro-CT images of operative lumbar intervertebral spaces in the 3 different procedures at 12 months postoperatively: **(D)** discectomy-only space (control group), **(E)** discectomy with autologous bone implanted space (autograft group), and **(F)** discectomy with porous tantalum implanted space (tantalum group). Both the autograft and tantalum groups developed solid fusion with a continuous bony bridge from the cranial to the caudal vertebra, while non-fusion was observed in the control group. **(G)** The imaging fusion index scores at different postoperative time points. Stained non-decalcified sections showing new bone formation associated with osteonecrosis in the autograft **(H)** and porous tantalum interface **(I)** groups (Lu et al., 2019).

Tantalum has been increasingly used in the field of orthopedics because of its good histocompatibility, strong corrosion resistance, and so on (Zardiackas et al., 2001; Veillette et al., 2006). Lu *et al.* demonstrated through *in vitro* and *in vivo* experiments that porous tantalum implants can achieve good fusion, and tantalum is expected to be an effective biomaterial for interbody fusion cages. Porous tantalum has a structure and mechanical properties to similar to those of human bone, allowing trabecular bone to grow into the pores of a cage implant to achieve better osseointegration. In addition, tantalum stimulates cell proliferation and enhances the osteogenic capacity of human osteoblasts, improving vertebral fusion efficiency (Lu et al., 2019) (Figure 3). Above all, compared to autogenous iliac bone graft, the adverse events in donor site can be avoided. On the other hand, tantalum has been reported in the treatment of infectious bone defects and bone tumors, which can be used in interbody fusion and spinal metastases after vertebrectomy (Hua et al., 2020; Wang J et al., 2020). At present, there are few reports about tantalum interbody fusion cages in the clinic and experiments, and its effect and characteristics need to be further verified (Iv, Beutler et al., 2010).

### PEEK

PEEK is a kind of high-molecular-weight semi-crystal polyaromatic linear polymer and thermoplastic material characterized by biological inertia, radiation transmission, low density, strong corrosion resistance, and a bone-like hardness (Basgul et al., 2019). Compared to alloy, PEEK can minimize stress shielding and bone resorption around the implant to avoid implant loosening (McGilvray et al., 2018; Chu et al., 2019). However, the formation of a biofilm on the surface of PEEK inhibits its binding to the host bone, preventing solid fusion (McGilvray et al., 2017; Gunzburg et al., 2019). In addition, PEEK cages can cause local inflammation, which may lead to bone non-union and osteolysis (Mokawem et al., 2019; Cheng et al., 2020). In recent years, the technology for 3D-printing PEEK interbody cages has matured, and there have been reports of its successful clinical application. Because of PEEK high melting point, laser sintering is commonly used, and the mechanical properties, biocompatibility, and heat resistance of PEEK can be effectively improved by adding in other inorganic materials and polymers (Tan et al., 2005). It is reported that the fusion rate of PEEK interbody devices is similar to that of allogeneic bone grafts (Jain et al., 2020).

**FIGURE 4:**
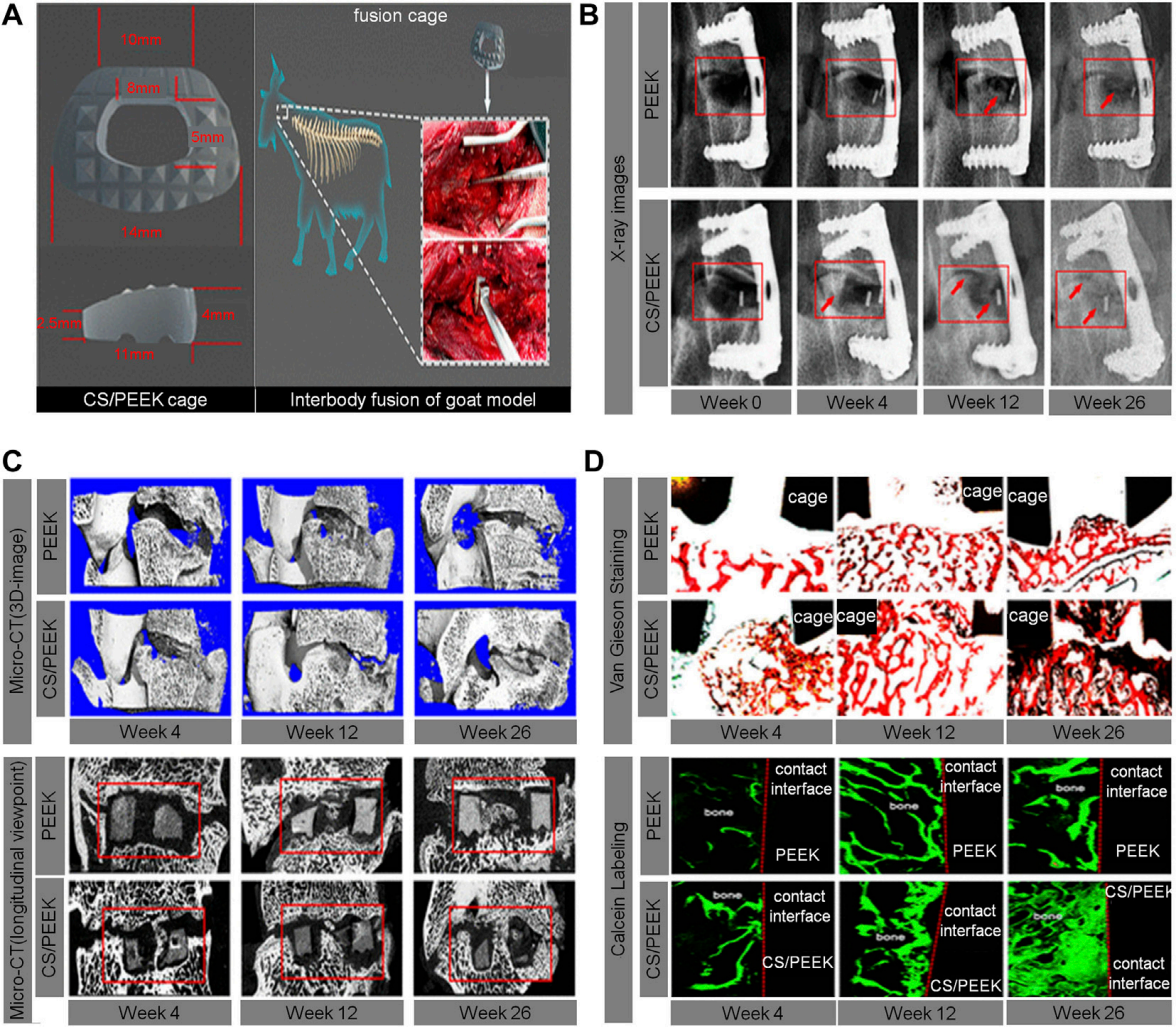
**(A)** The size and implantation of the CS/PEEK cage in a goat interbody fusion model. **(B)** Interbody fusion at 0, 4, 12, and 26 weeks based on the X-ray observation; representative X-ray images with red arrows indicate the bone ingrowth. **(C)** 3D and longitudinal images of the interbody fusion of bone formation in the interbody fusion at 4, 12, and 26 weeks based on the micro-CT scans. **(D)** Analysis of osseointegration at 4, 12, and 26 weeks based on histological assessment, including Van Gieson staining and fluorescence labeling by calcein (Chu et al., 2019).

The calcium silicate (CS)/PEEK composite used by Chu *et al.* was prepared by infusing CS into PEEK through compounding and injection-molding techniques. According to the mechanical property test results, the best ratio of CS is 40% (wt%). Through the analysis of the results from a goat interbody fusion model, it was found that the CS/PEEK composite had good biomechanical stability, osteogenic effects, and osseointegration ability. This bioactive material has great potential to facilitate the development of clinical interbody fusion cages (Chu et al., 2019) (Figure 4).

**FIGURE 5:**
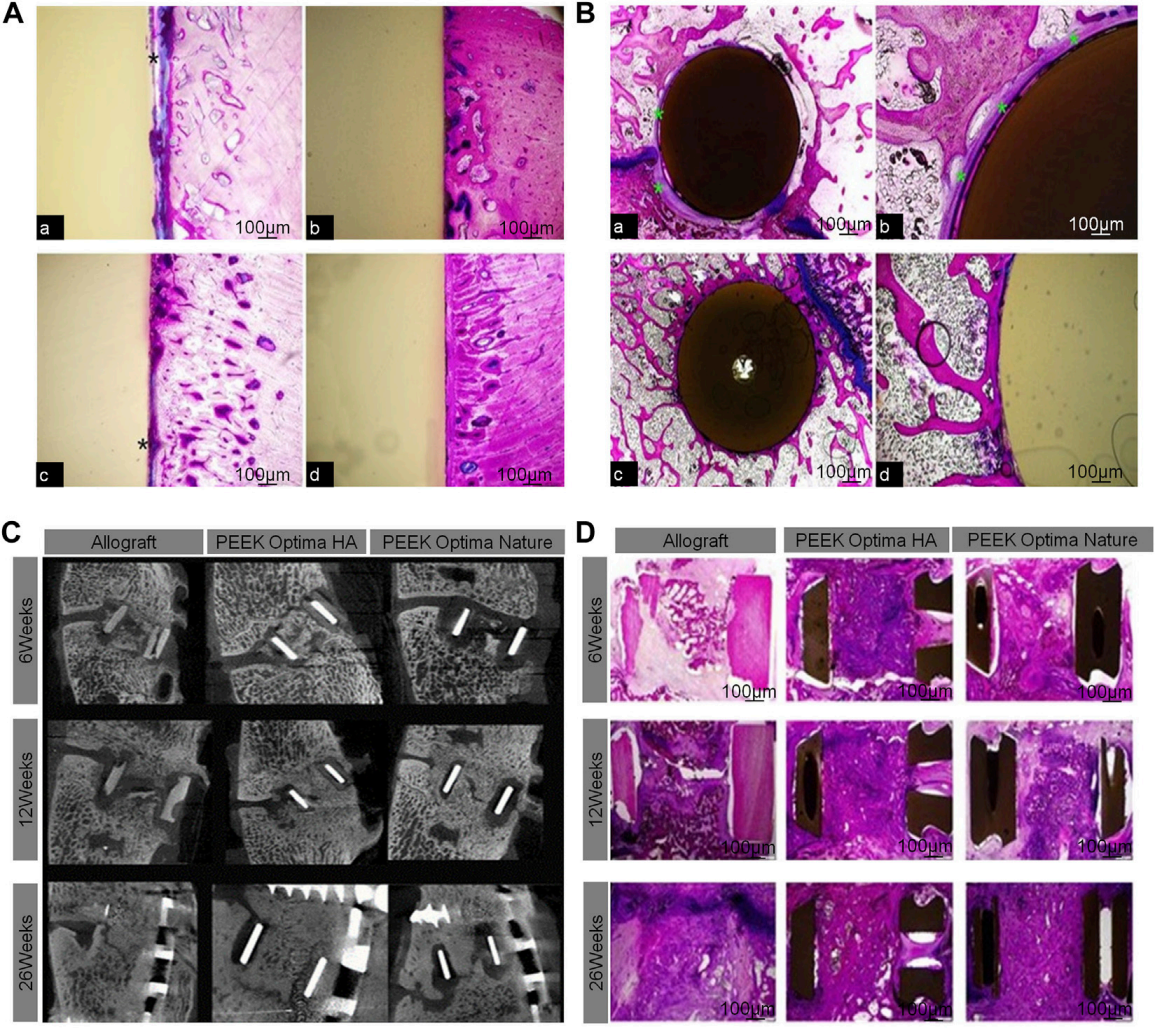
**(A)** Bone ingrowth in cortical sites for PEEK cage **(A,C)** and PEEK-HA cage **(B,D)** at 4 and 12 weeks demonstrated the presence of a fibrous tissue interface for PEEK (*), whereas a direct bone-to-implant interface was observed for PEEK-HA at the magnification used. **(B)** Bone ingrowth in cancellous sites for PEEK **(A,C)** and PEEK-HA **(B,D)** at 12 weeks demonstrated the presence of a fibrous tissue interface for PEEK (*), whereas a direct bone-to-implant interface was observed for PEEK-HA at the magnification used. **(C)** Micro-CT at 6, 12, and 26 weeks for allograft, PEEKHA, and PEEK demonstrated progression in fusion versus time for all groups. **(D)** Macroscopic overview of PMMA histology (Walsh et al., 2016).

McGilvray *et al.* found that, compared to the standard PEEK stent, the range of motion of a PEEK–Ti composite stent made using a mixture of PEEK and Ti was significantly reduced, the hardness was significantly increased, and the fusion effect was better (McGilvray et al., 2017). Dai *et al.* confirmed in their study that a PEEK interbody fusion cage containing β-tricalcium phosphate was efficient for the treatment of cervical spondylosis (Dai and Jiang 2008). The elastic modulus of the PEEK interbody fusion cage is similar to that of vertebral cortical bone, which is beneficial for the dispersion of load and stress and greatly reduces the probability of the cage sinking (Lim et al., 2019). William *et al.* found that the introduction of HA into a scaffold made of PEEK matrix allowed for direct bone-attachment growth and exhibited better fusion in a goat cervical fusion model. Although PEEK cages with HA can improve interbody fusion, there are no clinical studies to support their use (Walsh et al., 2016) (Figure 5). Also, although PEEK interbody fusion cages are relatively mature structures, their composite materials need to be further explored.

### Absorbable Materials

Absorbable materials have relatively good biocompatibility and can be hydrolyzed or enzymatically hydrolyzed into non-toxic small molecules that can be absorbed by organisms, with some even participating in biological metabolism (Hojo et al., 2005). Polylactide (PLA), alkyl polyglycoside, and polydioxane are considered safe and sufficiently stable absorbable materials, while iron, Mg, and zinc are the main absorbable metal elements. Although the inconvenience of a second surgery can be avoided by harvesting biomaterials, the possible complications, such as soft-tissue reactions, unwanted osteolysis, and low primary mechanical loading capacity, also limit the use of absorbable interbody fusion cages (Ulum et al., 2019; Pisecky et al., 2021). The hardness of the bioabsorbable fusion cage is similar to that of bone, which may accelerate interbody fusion. Meanwhile, as the fusion cage degrades, the load is gradually transferred to the healed bone to aid with bone remodeling. At present, PLA is the most commonly used bioabsorbable scaffold material, but rapid degradation and osteolysis around the scaffold have been reported (Ren et al., 2017). In a previous study, Lu *et al.* compared a novel PLA/nanoscale β-tricalcium phosphate bioabsorbable self-retaining cervical cage (BCFC) with an autologous bone graft and PEEK cage for anterior cervical discectomy and interbody fusion and found that the BCFC device had better fusion than the autologous bone graft or PEEK cage. This device may be a potential alternative to current PEEK cages (Lu et al., 2017). Knutsen *et al.* designed a bioabsorbable polycaprolactone (PCL) cervical cage and tested its compression, compression-shear, and torsion, concluding that PCL bioabsorbable cages may require additional fixation to achieve the effect of fused vertebral bodies (Knutsen et al., 2015). Through a clinical comparison, Jiya *et al.* found that absorbable poly-L-lactide-co-D,L-lactide (PLDLLA) implants are less effective in assisting with spinal fusion than PEEK implants, have a lower fusion rate, and are more prone to subsidence and osteolysis, so their benefits need to be further proven. The sterilization of PLDLLA cages by electron beam irradiation may lead to preliminary cage rupture, which is the main cause of mechanical failure (Jiya et al., 2009; Jiya et al., 2011). Ren *et al.* evaluated the fusion effect, biomechanical stability, and histological characteristics of a novel absorbable poly–amino acid copolymer/nano-hydroxyapatite/calcium sulfate (MAACP/HA/CS) interbody fusion cage in a goat cervical fusion model. Compared to a Ti cage and autogenous bone graft, the MAACP/HA/CS cage had a better fusion effect, combined closely with the surrounding bone, and maintained an appropriate intervertebral disc height (Ren et al., 2017). Although the absorbable material interbody fusion cage attained a good fusion rate because of its excellent biocompatibility and easy absorption, given its high failure rate, its clinical application needs to be further explored and studied.

Mg has a similar mechanical behavior to natural bone, good bone conduction activity, and radiation transparency, so it is widely regarded as a potential ideal absorbable orthopedic material superior to traditional metal and other biodegradable implants (Wang Y et al., 2020). The density of Mg and its alloy is close to that of human bone mineral, and its elastic modulus is about 45 GPa, which is lower than that of Ti_6_Al_4_V, tantalum, and other implantable metals (Staiger et al., 2006). Furthermore, it has been shown that implant-derived Mg induces local neuronal production of calcitonin gene–related polypeptide-α to improve bone-fracture healing (Zhang et al., 2016). The Mg^2+^ released from the degradation of Mg-based implants is considered an effective material to promote osteogenesis, but there are few studies on Mg-based implant fusion. Through degradation, interbody fusion, and biocompatibility testing of a goat cervical vertebra model, Guo *et al.* found that Mg-based interbody fusion cage had better histological fusion, but the total fusion area needed to be improved. This is the first report of successful histological fusion of a Mg-based interbody fusion cage (Guo et al., 2020). The main obstacle to limiting the clinical application of Mg-based cages is the adverse reactions caused by the rapid degradation of Mg. The degradation rate is faster in the first 3 weeks after implantation, then slows down gradually thereafter. However, excessive Mg accumulation caused by rapid implant corrosion will lead to a severe foreign body reaction, tissue stimulation, decreased mechanical strength of new bone, and abnormal precipitation of calcium, which will eventually hinder osteogenesis. Moreover, Mg generates a large amount of hydrogen during the corrosion process, resulting in tissue loosening or excessive pressure, which is not conducive to bone formation (Wessels et al., 2012). This may be because Mg scaffolds corrode faster than new bone formation, leading to local scaffold collapse, resulting in differences in intervertebral pressure and ultimately leading to fusion failure (Staiger et al., 2006). In an experiment of a silicon-coated Mg alloy (AZ31) fusion cage in a goat cervical vertebra model, Zhang *et al.* found that excessive Mg accumulation could inhibit the formation of new bone, the intervertebral Mg ion concentration increased significantly after implantation, and the intervertebral Ca/P ratio decreased under the condition of high Mg accumulation, which may be the main reason for the failure of interbody fusion (Zhang F et al., 2018). However, there is only a single case of successful fusion in the literature; moreover, even after modification to balance the degradation rate, the fusion region is still too small to meet clinical needs, so more research is needed to improve it. It is a feasible method to slow down the degradation rate of Mg by reducing the purity of Mg (Chang et al., 2020). At present, Mg as an intraspinal plant is still in an exploratory stage, and its clinical application requires more experimental support.

### Ceramic Materials

Bioceramics have inorganic components similar to natural bone which are widely used for bone tissue scaffolds fabricating. Due to its similar composition to natural bone, bioceramics present a significant osteogenic differentiating capability (Dxa et al., 2020). Bioceramic materials consist of calcium phosphate ceramics (CaP) such as hydroxyapatite (HA), tricalcium phosphate (TCP), and bioglass (Zhao et al., 2021). It is known that the biologically active CaO-SiO_2_-P_2_O_5_-B_2_O_3_ glass–ceramic can chemically combine with bone to form a carboxyapatite layer to promote the differentiation of human bone marrow mesenchymal stem cells into osteoblasts. Li *et al.* found that the fusion rate and improvement of clinical symptoms of CaO-SiO_2_-P_2_O_5_-B_2_O_3_ glass–ceramic spacers were similar to those of Ti cages. In an *in vivo* model, cylindrical implants showed better osseointegration with adjacent bones than HA, and the surface coating was found to improve the osseointegration of the implant, while its compressive and flexural strength were ≥2 times that of HA alone. The compressive strength of CaO-SiO_2_-P_2_O_5_-B_2_O_3_ glass–ceramics is 4 times that of PEEK and 1.3 times that of Ti, respectively. However, this increased mechanical strength correlates with a greater risk of sinking in patients with osteoporosis (Lee et al., 2016). Ceramic implants made of silicon nitride show better biocompatibility and bone conductivity and are expected to reduce complications like subsidence and displacement. Kersten *et al.* compared a silicon nitride interbody fusion cage and PEEK interbody fusion cage in patients with symptomatic degenerative lumbar disc disease for the first time, but their results have not yet been published (Kersten et al., 2014). HA is a widely used ceramic biomaterial because it is the basis of bone tissue. In addition, it also provides enough matrix for the endogenesis of tissue in the process of bone regeneration. It generally needs to be used in combination with other materials. When the scaffold is made of chitosan and HA in a ratio of 20–80, scaffold has both elasticity and an osteogenic ability. In a mouse spinal fusion model, a chitosan/HAp composite scaffold showed good biocompatibility and sufficient spinal stability and induced solid and well-structured bone regeneration (Rodriguez-Vazquez and Ramos-Zuniga, 2020). In addition, a hollow HA/polyamide 66 stent (HA/PA66) has been used in anterior cervical reconstruction. Liang *et al.* verified in a goat cervical fusion model that porous HA/PA66 with a 300-μm pore size is more conducive to bone growth and can more effectively promote interbody fusion, providing a potential application prospect for intervertebral reconstruction after vertebrae resection (Liang et al., 2020).

## 4 Post-processing Techniques

### Coating

Coating technology is also widely used in orthopedic implant modification. The coating of the cage can modify the surface of the material to amend and increase its surface roughness without changing its biomechanical properties. Coating an interbody cage reduces the vertebral body subsidence and pseudoarthrosis (Rao et al., 2014; MacBarb et al., 2017). Coating techniques, including plasma spraying, chemical vapor deposition, metal–organic chemical vapor deposition, electrochemical vapor deposition, molten coating, physical vapor deposition, thermal or diffusion conversion, and sol-gel, have been used to deposit micron and nanometer coatings on various substrates, including Ti and its alloys. Nano-coating technology refers to thin films with thicknesses of 1–1,000 nm or those less than micro-coatings (<1 μm) (Choi et al., 2020). Here, we will introduce the coating techniques commonly used in interbody fusion.

**FIGURE 6:**
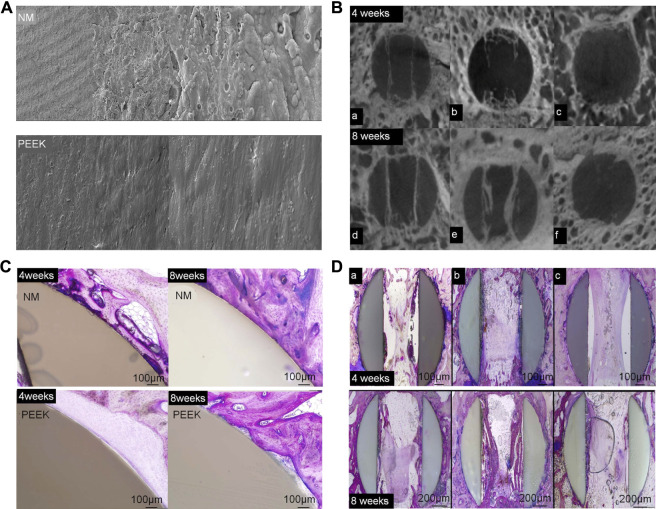
**(A)** Scanning electron microscopy was used to examine the surface topography of NanoMetalene (NM) and PEEK at magnifications of ×500, 5,000×, 20,000×, and 50,000×. **(B)** Micro-CT image reconstructions in the middle of an aperture at 4 and 8 weeks in the sagittal plane for each group. Group 1 was coated with NM on all surfaces, Group 2 had a NM coating inside the apertures with a PEEK outside, and Group 3 had PEEK with no coating. **(C)** A typical higher-magnification visual of the outside of the NM-coated surface and PEEK implants at 4 and 8 weeks. Direct bone contact with the Ti coating with NM occurred at 4 weeks, which improved over time to 8 weeks. PEEK surfaces presented a typical non-reactive fibrous tissue interface at 4 weeks, with some focal bone contact at 8 weeks. **(D)** PMMA histology in the middle of the apertures at 4 and 8 weeks in the sagittal plane (Walsh et al., 2018).

Gunzburg *et al.* verified that a PEEK material coated with nano-Ti can achieve greater spinal stiffness than a pure PEEK material in spinal fusion using a goat model (Gunzburg et al., 2019). The Ti coating on PEEK retains the physical properties of the PEEK substrate and contains a surface that reacts better with the host. Ti-surfaced PEEK implants allow for earlier and better fusion of new bone formation and remodeling by increasing the anchorage of osteoblasts in the endplates and wells. The Ti coating aids in bone formation and remodeling. This is accomplished by stimulating the production of osteoprotegerin, transforming growth factor (TGF)-β1, vascular endothelial growth factor (VEGF) A, fibroblast growth factor (FGF), and angiopoietin 1. Local levels of these factors were higher in Ti-coated implants than smooth Ti_6_Al_4_V or PEEK alloys (Bassous et al., 2019; Ohanisian and Dorsi 2019). In a prospective evaluation of 45 patients undergoing ACDF, Krayenbuhl *et al.* achieved good interbody fusion with a low complication rate by placing an empty plasma hole–covered Ti cage after anterior cervical microsurgery. Disc height and lordosis can be preserved with a low incidence of subsidence and good fusion rates (Krayenbühl et al., 2008). Walsh et al. (2018) found that newly formed bone grew along the surface of a nano-Ti–coated PEEK fusion cage in a sheep model. On the other hand, the surface of PEEK cage shows non-reactive fiber tissue at the interface (Figure 6). The coated PEEK fusion cage had good adhesion and proliferation of bone marrow stromal cells (BMSCs) and can induce a higher cell growth rate and alkaline phosphatase level, which can promote bone growth (Liu et al., 2017).

**FIGURE 7:**
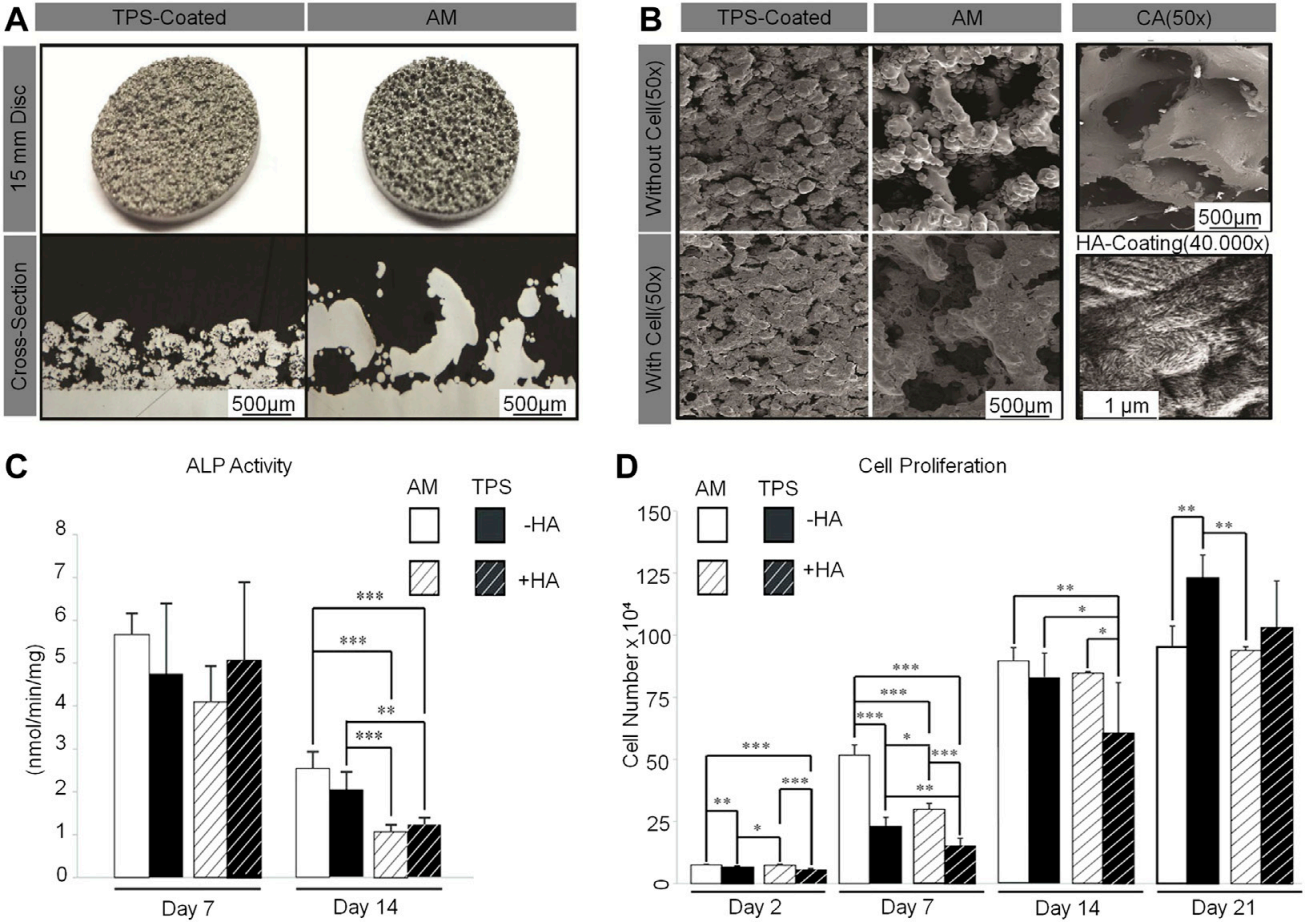
**(A)** Gross images of TPS-coated and additive-manufactured (AM) discs (top). Cross-section images taken from stereological analysis (bottom). **(B)** Representative scanning electron microscopy images of unseeded TPS-coated and AM discs (top left). Discs after 3 weeks of culture with human 1.19 fetal osteoblast-like (hFOB) cells (bottom left) and of human cancellous bone allograft to show the target surface topography (top right). Representative scanning electron microscopy images of nano-crystalline HA coating on an AM disc (bottom right). **(C)** Two-way analysis of variance of AM compared to TPS-coated discs with and without a nano-crystalline HA coating for alkaline phosphatase activity. **(D)** Two-way analysis of variance of AM compared to TPS-coated discs with and without a nano-crystalline HA coating for cell proliferation (MacBarb et al., 2017).

The common method to enhance osteoblasts’ response to a material is to apply HA to the surface of cage (MacBarb et al., 2017; Walsh et al., 2018). Sierra *et al.* 3D-printed a lumbar cage with HA coating and found that it had good biocompatibility through *in vitro* analysis, characterization, and testing (Serra et al., 2016). McBarb *et al.* designed and compared Ti plasma spray (TPS) coatings with and without nano-crystalline HA coatings. Their *in vitro* experiments showed that the HA coating achieved better cell proliferation and osteogenic capacity compared to the uncoated TPS, but the difference was not statistically significant (MacBarb et al., 2017) (Figure 7).

The natural extracellular matrix protein is considered a candidate material for the development of biomaterials that can induce specific cell behaviors. As a surface coating of biomaterials, the purified protein can change the proliferation and differentiation of stem cells and the retention of growth factors, which provides a new idea for the coating technology of printing materials. This could be explored in future experiments (Xing et al., 2020).

### Internal Fillings

Obtaining good biocompatibility of fillers through internal filling of stents, promoting local proliferation, and obtaining a good osteogenic ability to promote interbody fusion are commonly used methods in basic experiments and clinical practice of spine surgery. Additives such as drugs, growth factors, and platelet-rich plasma (PRP) are added to the pre-designed porous interbody fusion cage by means of negative pressure suction, direct filling, and immersion (Bai H. T. et al., 2020; Leng et al., 2020).

#### 4.2.1 Drugs

##### 4.2.1.1 Simvastatin

**FIGURE 8:**
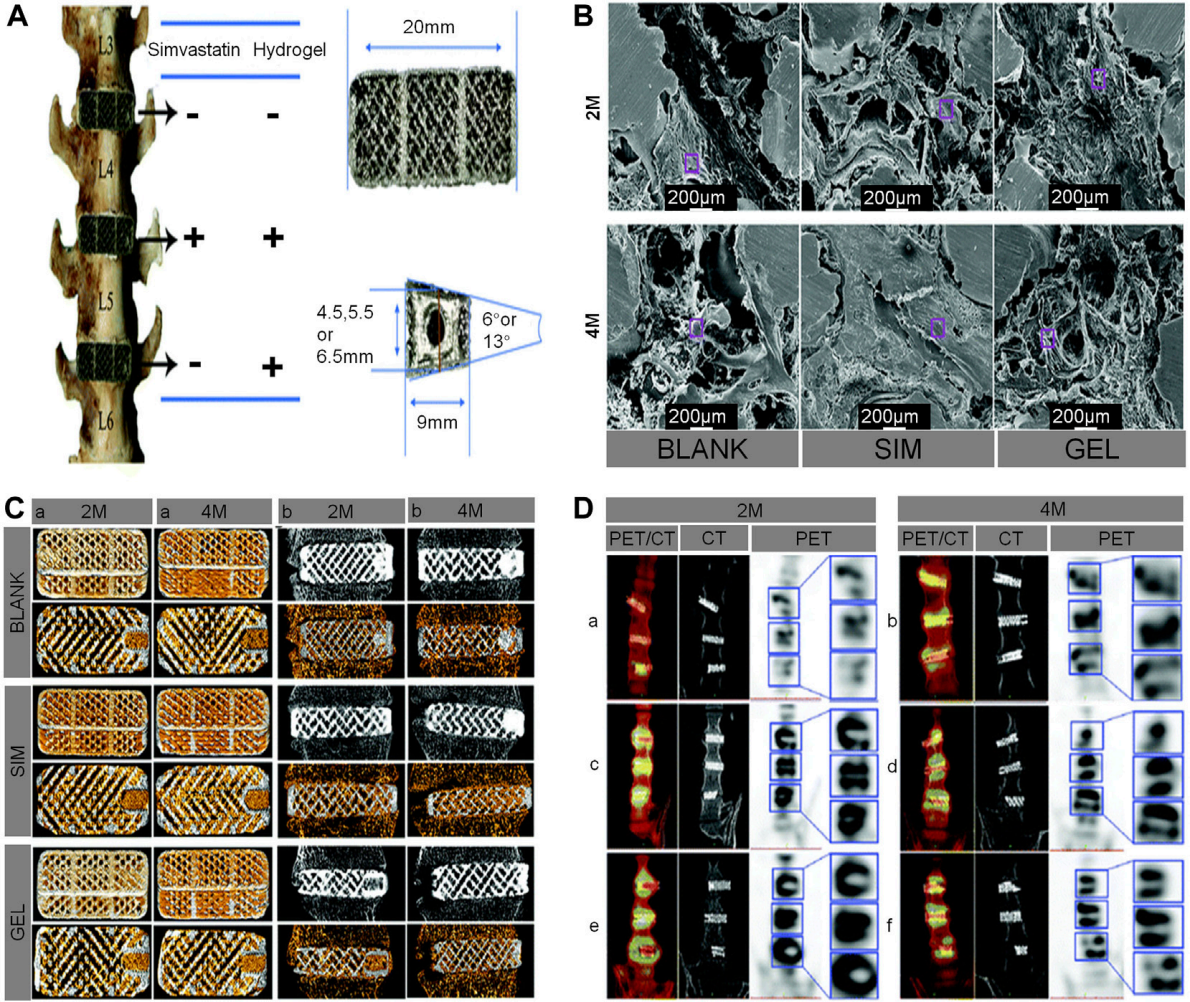
**(A)** The distribution of the cages in the lumbar interbody spaces, the geometrical parameters of the cages, and a screw hole included on one end of the cage. **(B)** Characterization of the intra-porous morphology in cages using scanning electron microscopy, which showed that the bone formation in the cages of the SIM group was very dense. **(C)** Bone ingrowth in the cages (yellow represents new bone) at 2 and 4 months. For each cage in the different fused segments, the upper panel shows 3D-reconstructed images of the cages, and the lower panel shows axial 2-dimensional images of the cages **(A)**. Osseointegration around the cages (gray in the upper panel and yellow in the lower panel both represent new bone in cages) **(B)**. **(D)** Coronal fused positron emission tomography/CT, CT, and positron emission tomography and a locally enlarged image of the macaques at 2 and 4 months, respectively (Zhang et al., 2020a).

Over the years, statins have been widely used to lower cholesterol and reduce the risk of heart attack (Song et al., 2003). SIM, an inhibitor of 3-hydroxy-3-methylglutaryl coenzyme A reductase (HMG-CoA), which has the advantages of a low price, good safety, and ability to promote bone growth in a prosthesis (Jin et al., 2021). SIM overcomes the shortcomings of the high cost and short half-life of biological factors like BMP-2 and VEGF used in previous studies (Zhang et al., 2020a). Topical application of SIM has a good effect on bone formation. Moreover, local application of PLA has achieved good results in experiments of bone formation promotion and bone defect healing (Zhao et al., 2014; Tan et al., 2015). Using porous Ti cage filled with SIM/poloxamer 407 hydrogel in a rhesus monkey interbody fusion model, Zhang *et al.* found that 3D-printed porous scaffolds containing SIM hydrogel promoted bone growth and spinal fusion. They also speculated that, in the early stage of osteogenesis, the continuous release of SIM from the hydrogel promotes the production of endogenous biological factors, such as BMP-2 and VEGF, which are key regulators of bone formation and angiogenesis during bone regeneration. However, with the passage of time, due to the body’s metabolism, the osteogenic effect of SIM gradually decreases (Zhang et al., 2020b) (Figure 8).

##### 4.2.1.2 Strontium Ranelate

SRR is an oral drug used for the prevention and treatment of osteoporosis. Different from other anti-osteoporotic drugs, SRR has unique dual effects on bone formation and bone resorption. At the same time, it reduces bone decomposition, stimulates bone reconstruction, prevents bone loss, improves bone strength, and reduces the risk of fracture (Rodrigues et al., 2018; Demirel and Kaya 2020). However, oral SSR may have no synthetic metabolic effect on human bone formation et al., 2018, Marx et al., 2020). Tai *et al.* found that SRR treatment had a relatively mild effect on bone strength and bone remodeling and confirmed its better interbody fusion through histological and mechanical tests in a mouse interbody fusion model, suggesting that SRR treatment can be used as a replenish for human interbody fusion, although an interbody fusion cage was not used in this mouse model. This experiment provides a new idea for interbody fusion under the umbrella of osteoporosis (Tsai et al., 2017).

#### 4.2.2 Platelet-Rich Plasma

PRP obtained by centrifugation is a plasma product containing high concentrations of various growth factors, including platelet-derived growth factor, insulin-like growth factor (IGF), and TGF-β. PRP is known to play an important role in fracture healing. A large number of studies have shown that PRP-modified scaffolds have a positive effect on bone regeneration and can significantly promote the repair of bone defects (Lowery et al., 1999; Carreon et al., 2005; Leng et al., 2020; Sochacki et al., 2020; Andrade et al., 2021). The application of PRP in a rat spinal fusion model significantly accelerated the speed of interbody fusion without any complications (Shiga et al., 2016). In addition, lyophilized PRP is also widely used due to its advantage of easy storage. Compared to traditional PRP, lyophilized PRP–coated Ti more strongly promoted the cell viability and osteogenic differentiation of BMSCs and triggered better bone formation. Interbody fusion provides a new idea (Qiao et al., 2020). It can be seen from previous literature that there are no animal experiments of interbody fusion cages combined with PRP, but PRP’s role in promoting interbody fusion has been tested alone, which provides a direction for future experiments and research.

#### 4.2.3 Growth Factors

Soluble growth factors obtained by protein separation and molecular cloning techniques include TGFs, BMPs, IGFs, FGFs, and epidermal growth factor. However, only some of these growth factors showed a significant bone-induction ability (Kandziora et al., 2003).

##### 4.2.3.1 Bone Morphogenetic Protein

**FIGURE 9:**
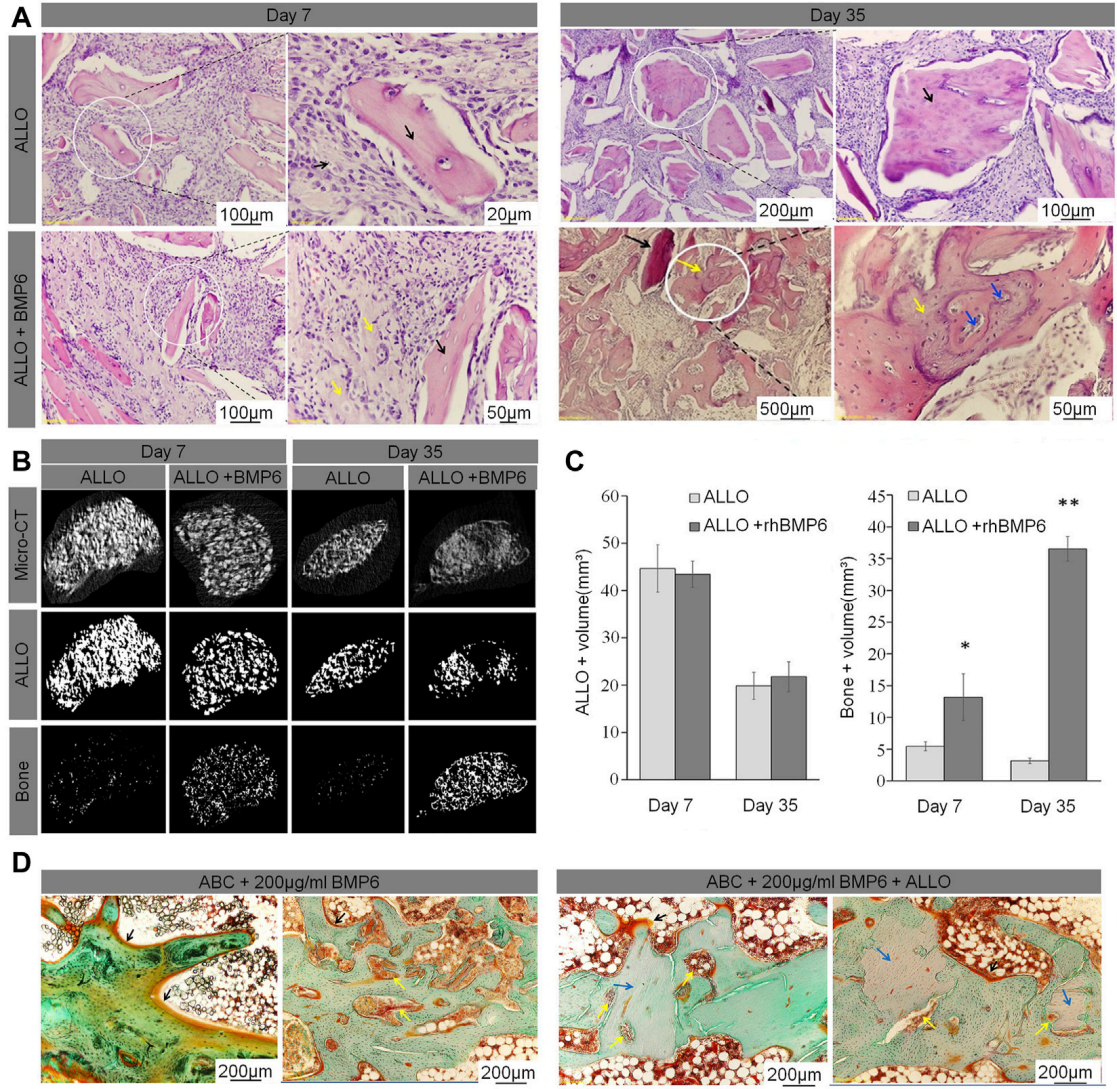
**(A)** ABC/allograft devitalized bone particles (ALLO) (black arrows) without RhBMP-6 induced formation of fibrotic tissue at days 7 and 35 without any sign of new bone formation. ABC/ALLO with RhBMP-6 had induced new bone formation by day 7 (yellow arrows), and advanced creeping substitution of ALLO with new bone was observed on day 35 (blue arrowhead). **(B)** Overall micro-CT analyses of ABC/ALLO implants without and with RhBMP-6 are shown in the top row, only ALLO particles are visualized in the middle row, and the bottom row shows the newly formed bone (images obtained upon subtracting the ALLO particles from overall micro-CT images), respectively. Note the formation of new bone by day 7, with significant formation by day 35 in ABC/ALLO implants that contained RhBMP-6. ABC/ALLO alone did not induce bone either at 7 or 35 days after implantation. **(C)** Morphometric analysis of ALLO volume in implants on days 7 and 35, indicating a significant decrease in ALLO volume and increased amount of bone in the presence of RhBMP-6. **(D)** Histology of ABC implants without and with ALLO harvested at 14 weeks after surgery in a rabbit model. Note the newly formed bone with a dense trabecular structure with laid-down osteoids at the surface (black arrows), pronounced bone remodeling with numerous blood vessels (yellow arrows), and the newly formed trabeculae assimilated with ALLO (blue arrows) (Grgurevic et al.).

BMP has attracted wide attention because of its inherent potential to improve the process of ossification and induce pluripotent progenitor cells to differentiate along the direction of osteogenesis. RhBMP-2 is an osteoinductive protein that has been used to promote spinal fusion and can induce fusion when used as an implant with a suitable carrier, such as an absorbable sponge (Boden et al., 2000; Burkus et al., 2017). BMP acts as a signal to stimulate BMSCs to migrate to the implantation site and induces marrow stromal cells (MSCs) to proliferate and differentiate into osteoblasts (Grgurevic et al., Bae et al., 2016). Previous studies on BMP-2 engraftment have shown that it has many effects, including angiogenesis, cell recruitment, and even promotion of mesenchymal and hematopoietic progenitor cell proliferation. However, new research suggests that BMP behaves differently at different doses (Burkus et al., 2002; Meisel et al., 2008). By adding RhBMP-2 to the fusion cage, Pan *et al.* found that, although BMP accelerated the speed of interbody fusion, it also produced a significant inflammatory response. In clinical applications, BMP may lead to local adverse structural changes and eclectic bone biomechanical changes, osteolysis, and trabecular thinning (Pan et al., 2017). Sethi *et al.* found that the use of RhBMP-2 can increase the fusion rate, but the incidence of pre-vertebral soft tissue swelling also increases (Sethi et al., 2011). The most common adverse reactions include accidental swelling and inflammation as well as inflammatory cyst formation. Although there is no difference in clinical effect between interbody fusion and autograft placement, BMP can also lead to endplate absorption, sinking, increased incidence of cage migration, osteolysis, and ectopic bone formation (Sethi et al., 2011; Michielsen et al., 2013; Burkus et al., 2017). Use of RhBMP-2 is strictly limited in clinical applications. It is relatively effective in achieving bone fusion, and the fusion rate is similar to that of patients receiving autografts. The incidence of complications was similar between autografts and BMP, but the rates of radiculitis and serum tumor were slightly higher in the BMP group (Khan et al., 2018). There was no significant difference in fusion rate between low and high doses (low: 33.3% and high: 46.7%), but a high dose could easily lead to an increased incidence of osteolysis, adverse events, and swelling (Okada et al., 2020). During a postoperative evaluation of 17 patients with lumbar degenerative disease after implantation of a PEEK interbody cage with 212 mg of a RhBMP collagen gel sponge, Meisel *et al.* found that there was no significant difference between the fusion rate and autologous bone graft, but there was temporary bone resorption around the fusion cage at 3 months without subsidence, pain, or complications (Meisel et al., 2008). In a sheep interbody fusion model, Bae *et al.* used an absorbable sponge with different doses of RhBMP-2 in a customized PEEK interbody fusion cage and found that the osteoclast activity and the corresponding peri-implant bone resorption rate were dose-dependent and reached a peak 4 weeks after operation (Bae et al., 2016). Grgurevic *et al.* induced complete spinal fusion with recombinant human RhBMP-6 based on an autologous blood coagulant (ABC) in sheep model. The best dose was 100 μg of RhBMP-6/mLABC (Grgurevic et al.) (Figure 9). An absorbable collagen sponge soaked in recombinant BMP-2 and recombinant BMP-7 combined with bovine collagen have been approved as substitutes for biological bone grafts to close gaps and repair delayed and non-union fractures. Among them, the incidence of complications in different positions is varied. Anterior cervical approach always causes the most serious side effects including anterior cervical edema, osteolysis, graft sedimentation and wound infection. Followed by posterior cervical approach which can cause posterior neck pain. The side effects in anterior and posterior lumbar approaches are relatively mild. Moreover, the tumorigenic effect of BMP should also be noted (Joseph and Rampersaud, 2007). In summary, BMP can significantly promote spinal fusion in the short term, but its side effects and dose sensitivity limit its wide application. Currently, BMP-2 has been proved by Food and Drug Administration (FDA) and used as an INFUSE Bone Graft (Medtronic Sofamor Danek) clinically. The recommended dosage varied between studies, and we draw a comprehensive conclusion among references that 1.5 mg/cm3 is deemed as an ideal effective concentration of BMP-2 (Sethi et al., 2011). Moreover, the following researches should continuously focus on the appropriate BMP dosage weighing the biosafety and effectiveness.

**SCHEME 1:**
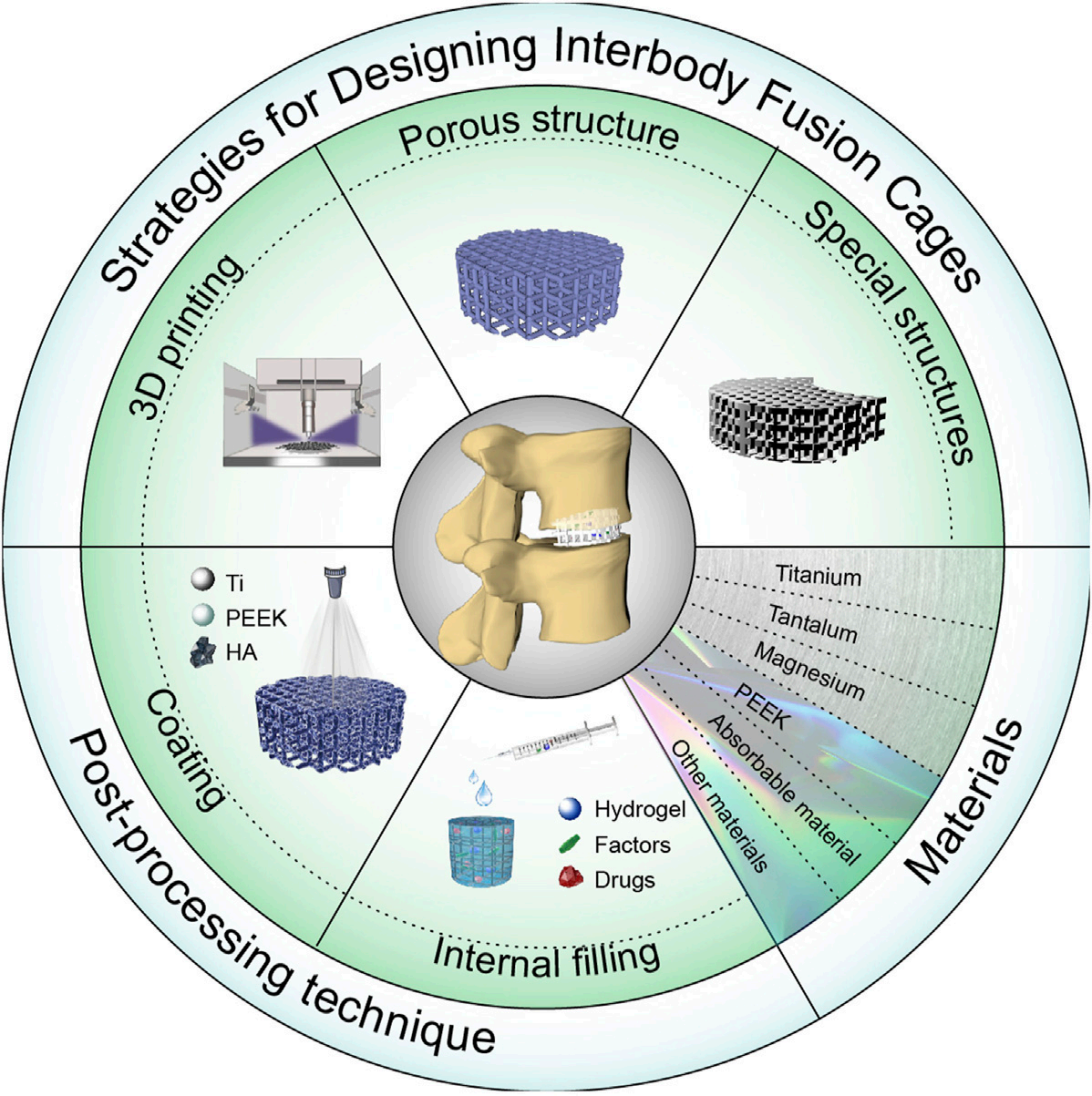
Schematic diagram of an interbody fusion cage. Design and application of the interbody cage.

##### 4.2.3.2 Combination of IGF and TGF-β

When the combination of IGF and TGF-β is used for spinal fusion, it can achieve mutual promotion. By adding IGF and TGF-β to HA-coated cages in a goat cervical fusion model, Gu *et al.* found the cage with IGF and TGF-β had significantly higher tensile strengths in extension and scoliosis compared to the other groups, indicating that they achieved better spinal fusion (Gu et al., 2013). Kim *et al.* used a fusion cage with IGF combined with TGF-β in a cervical fusion sheep model and found through quantitative CT that the fusion cage group with IGF combined with TGF-β showed significantly higher values for bone mineral density, bone mineral content, and bony callus volume at 12 weeks after implantation. This shows that these growth factors can improve the formation of the intervertebral bone matrix, but their long-term effects need to be further explored (Kim et al., 2015). By adding BMP and IGF/TGF-β into a Ti cage in a sheep cervical fusion model, Kandziora *et al.* found that the early fusion effects of BMP and IGF/TGF-β were similar. The callus in the BMP group and IGF/TGF-β group had higher bone mineral density and biomechanical stability during extension, rotation, and bending (Kandziora et al., 2002). This fully illustrates the strong potential of the combined use of IGF/TGF-β in spinal fusion and provides a strong basis for future clinical use.

#### 4.2.4 Allogeneic Material

Allogeneic cancellous bone, tricalcium phosphate, decalcified bone matrix (DBM), mineralized collagen, biphasic calcium phosphate, and other alternative materials have been successfully applied to the additives of interbody fusion cage, and good results have been obtained (Cho et al., 2005; Dai and Jiang 2008; Liao et al., 2008; Topuz et al., 2009; Scholz et al., 2010). Among them, CS is a kind of bone-substitute material. CS particles have been successfully used as implantable fillers in the treatment of periodontal bone defects and long bone defects (Thomas and Puleo 2010; Baranes and Kurtzman 2019). DBM has been proven to have the potential for bone conduction and bone induction because of the proteins and various growth factors present in the extracellular matrix. DBM alone can induce good bone bridging in lumbar interbody fusion, but the effect is still not as good as that of autogenous bone graft, so it is recommended that DBM be combined with other materials. Xie *et al.* found that, although the curative effect and fusion rate of a PEEK interbody fusion cage filled with CS/DBM and autogenous bone filler were similar, CS/DBM instead of autogenous bone had the advantages of less intraoperative blood loss and fewer complications at the donor site, so it is a good substitute for autotransplantation (Xie et al., 2015). Biomineralized collagen has a good osteogenic ability, and the osteogenic ability of adjacent bone surfaces has a profound clinical application value, showing a potential application prospect. In clinical cases, Barlocher *et al.* found that, compared to an autologous bone graft and threaded Ti cage, polymethyl methacrylate (PMMA) appears to be a good alternative to cage-assisted fusion. Although many complications caused by autologous transplantation are avoided, the inability to achieve bony fusion still limits its use (Bärlocher et al., 2002). Kong *et al.* created a new biomaterial mineralized collagen–PMMA bone cement by injecting mineralized collagen into PMMA. It was found that PMMA bone cement showed a good osteogenic ability in a sheep model, but this finding needs to be verified in further clinical transformational experiments. Minimally invasive injectable lumbar interbody fusion with mineralized collagen–PMMA bone cement has far-reaching clinical value and shows potential application prospects (Kong et al., 2020). However, not all allogeneic fillers are conducive to fusion. Cho *et al.* found that the fusion effect of a fusion cage containing biphasic calcium phosphate ceramic was lower than that of autogenous iliac bone graft and delayed the fusion period (Cho et al., 2005).

## 5 Interbody Fusion Cage Prospects

At present, there are many kinds of cages used in clinical and basic experiments that need to be chosen according to their respective advantages and needs. In this review, we list the advantages of different cage materials, internal implants, design methods, and post-processing to inform our use and selection.

In orthopedics clinics, the design of microporous fusion cages is particularly suitable for bone ingrowth and osseointegration, and the research on microporous structures is relatively mature. There are many studies on bone ingrowth with different porosities and pore sizes. Generally, the porosity is 65–80%, and its structure and mechanical properties are similar to those of trabecular bone. A pore size of 50–500 μm is conducive to the adhesion, proliferation, and differentiation of bone cells. With the innovation of additive manufacturing technology, production now offers unprecedented means of structuring and customization, and the complex anatomy of the spine is very suitable for patient-specific equipment. Improving implant designs through the 3D-printing process may improve osseointegration and reduce the sinking rate. Combined with the current, relatively popular approach of finite element analysis, the preliminary design can be established to analyze whether the elastic modulus under different porosities and pore sizes is similar to that of human bone so that better spinal fusion can be obtained. In addition, topoisomerism technology can render the contact area between the implant and bone larger, which will make bone ingrowth easier. At present, there is no interbody cage with a fixation device available in the implant itself, which is also a direction that can be explored. Combined with these technologies mentioned above, a relatively suitable interbody cage can be obtained before implantation, and then basic and clinical experiments are performed to verify the performance.

Nowadays, the 2 most commonly used materials of interbody fusion cages are Ti alloy and PEEK. While Ti alloy materials have good biocompatibility, their excessive strength is likely to cause fusion. Conversely, the strength of PEEK is similar to that of human bone, and it is often used because of the easy detection of transmitted rays, but its biological inertness limits its further use. However, by modifying the PEEK material or adding other compounds to print the cage, its biocompatibility has been improved. At present, the CS/PEEK cage has been proven to promote interbody fusion, but mixtures with other ceramic-like materials have not appeared, which provides a direction for future research. Tantalum, Mg, and their alloys and absorbable materials have relatively few applications, and their respective deficiencies limit their usefulness. In the future, a new Mg alloy will not fail due to rapid absorption and local collapse; instead, it will achieve a balance between the new bone and the Mg alloy scaffold to perfectly support the pressure between the vertebral bodies before eventually being perfectly replaced by the new bone. Other absorbable materials, such as PLA, can be modified to achieve a degree of compressive strength that can support intervertebral pressure, rendering this issue no longer a problem. In addition, copper alloys, cobalt alloys, and memory metals with antibacterial properties will provide new options for vertebral body fusion under different physiological conditions.

Transplantation of autologous bone and allogeneic bone is the most common method for interbody fusion, offering biocompatibility and no obverse adverse reactions. In addition to the above 2 materials, there are other internal plants and drugs that are conducive to osseointegration. Materials such as SIM, BMP, biomineralized collagen, and PRP all show good bone ingrowth induction function, but there are no precise data to guide their dosage, and further exploration and experiments are necessary. In the future, through research, there will be guidelines developed to standardize their use and dosage. In addition, it is known that, in the process of bone formation, different growth factors are required in different stages. In the future, different drug-release systems will be constructed, and different doses of drugs will be released at corresponding times to regulate intervertebral bone formation.

To sum up, interbody fusion is a process of bony fusion. The selection of materials and additives for general interbody cages serves to enhance their osteogenic properties, but, when designing an interbody cage, the developer must consider that the device needs to withstand the pressure from upper and lower vertebrae; otherwise, the collapse of the implant will lead to fusion failure.

## 6 Conclusion

Good selection and use of the interbody cage are essential in the prognosis of clinical spinal interbody fusion. However, there is still a shortage of systematic discussion of its usage to date. This review focused on the design, materials, and post-processing technologies of the interbody cage. 3D printing is commonly used in the design work, and the design of porous structures is conducive to the ingrowth of new bone, which is more effective in improving the fusion rate. Other special types of interbody cages have been designed to reduce the difficulty of surgery or to facilitate fixation. Ti alloy and PEEK are the more commonly used materials for interbody cages. Other materials are less frequently used; among them, Mg alloy is an absorbable material, and its rapid absorption limits its use. In addition, post-processing technology is also important, including coating technology and internal additives. Ti and HA coatings are more commonly used, and studies so far have found that coating technology can improve spinal fusion to a certain extent. Internal additives, including SIM, BMP, and PRP, can promote bone formation through their own metabolism; however, there are few related studies at present, which provides a direction for future research.
